# Investigating the efficacy of baricitinib in new onset type 1 diabetes mellitus (BANDIT)—study protocol for a phase 2, randomized, placebo controlled trial

**DOI:** 10.1186/s13063-022-06356-z

**Published:** 2022-05-23

**Authors:** M. Waibel, H. E. Thomas, J. M. Wentworth, J. J. Couper, R. J. MacIsaac, F. J. Cameron, M. So, B. Krishnamurthy, M. C. Doyle, T. W. Kay

**Affiliations:** 1 St Vincent’s Institute of Medical Research, Melbourne, Australia; 2 Department of Medicine, St Vincent’s Hospital, The University of Melbourne, Fitzroy, 3065 Australia; 3 Wentworth Department of Medical Biology, The University of Melbourne, Parkville, Victoria 3010 Australia; 4 The Royal Melbourne Hospital, Melbourne, Australia; 5 Robinson Research Institute, University of Adelaide and Women’s and Children’s Hospital, Adelaide, Australia; 6 St Vincent’s Hospital, Melbourne, Australia; 7 MacIsaac Department of Medicine, The University of Melbourne, Fitzroy, 3065 Australia; 8 The Royal Children’s Hospital, Melbourne, Australia; 9 The University of Melbourne, Melbourne, 3010 Australia

**Keywords:** Type 1 diabetes, Baricitinib, Janus kinase (JAK) inhibitor, Insulin, C-peptide, Randomized controlled trial (RCT), Autoimmune disease

## Abstract

**Background:**

Type 1 diabetes (T1D) places an extraordinary burden on individuals and their families, as well as on the healthcare system. Despite recent advances in glucose sensors and insulin pump technology, only a minority of patients meet their glucose targets and face the risk of both acute and long-term complications, some of which are life-threatening.

The JAK-STAT pathway is critical for the immune-mediated pancreatic beta cell destruction in T1D. Our pre-clinical data show that inhibitors of JAK1/JAK2 prevent diabetes and reverse newly diagnosed diabetes in the T1D non-obese diabetic mouse model. The goal of this study is to determine if the JAK1/JAK2 inhibitor baricitinib impairs type 1 diabetes autoimmunity and preserves beta cell function.

**Methods:**

This will be as a multicentre, two-arm, double-blind, placebo-controlled randomized trial in individuals aged 10–30 years with recent-onset T1D. Eighty-three participants will be randomized in a 2:1 ratio within 100 days of diagnosis to receive either baricitinib 4mg/day or placebo for 48 weeks and then monitored for a further 48 weeks after stopping study drug. The primary outcome is the plasma C-peptide 2h area under the curve following ingestion of a mixed meal. Secondary outcomes include HbA1c, insulin dose, continuous glucose profile and adverse events. Mechanistic assessments will characterize general and diabetes-specific immune responses.

**Discussion:**

This study will determine if baricitinib slows the progressive, immune-mediated loss of beta cell function that occurs after clinical presentation of T1D. Preservation of beta cell function would be expected to improve glucose control and prevent diabetes complications, and justify additional trials of baricitinib combined with other therapies and of its use in at-risk populations to prevent T1D.

**Trial registration:**

ANZCTR ACTRN12620000239965. Registered on 26 February 2020. ClinicalTrials.gov NCT04774224. Registered on 01 March 2021

## Administrative information

Note: the numbers in curly brackets in this protocol refer to SPIRIT checklist item numbers. The order of the items has been modified to group similar items (see http://www.equator-network.org/reporting-guidelines/spirit-2013-statement-defining-standard-protocol-items-for-clinical-trials/)

**Table:** 

Title {1}	A Phase 2, Randomised, Placebo Controlled Study Investigating the Efficacy of **Ba**ricitinib in **N**ew Onset Type 1 **Di**abe**t**es Mellitus (BANDIT).
Trial registration {2a and 2b}.	ACTRN12620000239965 [ANZCTR; registered 26/02/2020] NCT04774224 [ClinicalTrials.gov; registered 01/03/2021]
Protocol version {3}	Version 6 of 03-06-2021
Funding {4}	This research is funded by JDRF International, JDRF Australia the recipient of Australian Government Department of Health funding through the Emerging Priorities and Consumer-Driven Research Initiative, part of the Medical Research Future Fund (Grant ID: 4-SRA-2020-912-M-B), the National Health and Medical Research Council (NHMRC) of Australia, and the study sponsor, St Vincent’s Institute of Medical Research, Australia.
Author details {5a}	M. Waibel: St Vincent’s Institute of Medical Research, Australia. H.E. Thomas: St Vincent’s Institute of Medical Research, Australia. J.M. Wentworth: The Royal Melbourne Hospital, Australia. J.J. Couper: Robinson Research Institute, University of Adelaide and Women’s and Children’s Hospital, Adelaide, Australia. R.J. MacIsaac: St Vincent’s Hospital, Melbourne, Australia. F.J. Cameron: The Royal Children’s Hospital, Melbourne, Australia. M. So: St Vincent’s Institute of Medical Research, Australia. B. Krishnamurthy: St Vincent’s Institute of Medical Research, Australia. M.C. Doyle: St Vincent’s Institute of Medical Research, Australia. T.W. Kay: St Vincent’s Institute of Medical Research, Australia.
Name and contact information for the trial sponsor {5b}	Dr Michaela Waibel, Study Coordinator St Vincent’s Institute of Medical Research mwaibel@svi.edu.au Professor Thomas WH Kay, Coordinating Principal Investigator. St Vincent’s Institute of Medical Research tkay@svi.edu.au
Role of sponsor {5c}	This is an investigator initiated clinical trial. Study sponsor and investigators are fully responsible for design of the study and collection, analysis, and interpretation of data and in writing the manuscript.

## Introduction

### Background and rationale {6a}

#### Summary

Type 1 diabetes (T1D) places an extraordinary burden on individuals and their families, as well as on the healthcare system. It involves a lifelong need for insulin, along with frequent blood glucose measurements and strict lifestyle regulation. Despite major advances in insulin delivery and continuous glucose monitoring, controlling blood glucose within the normal range is still difficult. T1D is associated with the risk of potentially life-threatening complications, including those which are acute (hypo- or hyperglycaemia and ketoacidosis) and long-term (vascular complications affecting the heart, feet, eyes, kidneys and nerves).

The JAK-STAT pathway is critical for the immune-mediated pancreatic beta cell destruction in T1D. Our pre-clinical data show that inhibitors of JAK1/JAK2 prevent diabetes and reverse newly diagnosed diabetes in the non-obese diabetic (NOD) mouse model of T1D [1, 2]. The goal of this study is to investigate whether the JAK1/JAK2 inhibitor, baricitinib, has activity against the disease process involved in human T1D with the aim of preventing immune-mediated destruction of insulin-producing beta cells.

#### Scientific rationale and aim

In mouse models and humans with T1D, there is evidence that autoreactive T cells destroy the insulin-producing beta-cells in the pancreatic islets [3]. Pro-inflammatory cytokines play an important role in this response to amplify the response and make beta cells more susceptible to immune recognition [4–7].

The JAK-STAT signalling pathway is downstream of type I and II cytokine receptors. When cytokines bind to their receptor, specific receptor-associated Janus kinases (JAKs) are activated by phosphorylation. JAK molecules phosphorylate cytokine receptors, allowing them to selectively bind STAT (signal transducers and activators of transcription) family proteins—proteins that regulate gene expression [8]. A meta-analysis of gene and protein interaction networks amongst 10 different autoimmune diseases showed JAK-STAT and interferon (IFN) signaling as key converging pathways in autoimmune diseases [9]. While STAT1 knockout NOD mice are protected from diabetes [10], blockade or deletion of individual cytokines or their receptors has much less impact on disease [3, 5, 11]. This underscores the rationale for manipulating cytokine signaling pathways rather than specific cytokines.

JAK inhibitors act on T cells and beta cells making them highly attractive candidate therapies for T1D. Furthermore, large clinical trials primarily in rheumatological diseases have shown that baricitinib, and the class of drugs more generally, is effective [12, 13] and well-tolerated [14, 15]. Our lab has successfully employed JAK1/JAK2 inhibitors to reverse established autoimmunity in NOD mice [1, 2]. JAK inhibitor exposure leads to the downregulation of MHC class I on beta cells, reduction in proliferation of islet antigen-specific T cells, reduction in insulitis and prevention of beta-cell loss [2].

The aim of this study is to assess whether the JAK inhibitor baricitinib can slow the progressive, immune-mediated loss of beta cell mass and function that occurs after clinical presentation. This will be measured via plasma C-peptide, a surrogate marker of insulin secretion.

#### Clinical experience with JAK inhibitors

Cytokine inhibition has revolutionized the treatment of autoimmune and inflammatory diseases through monoclonal antibodies that target specific cytokine pathways such as TNF, IL-1, IL-6 and more recently IL-23/IL-12 [16]. JAK inhibitor drugs were introduced through the discovery of tofacitinib, a drug that targets the ATP-binding pocket of JAK3 [17]. Tofacitinib has become a treatment for rheumatoid arthritis and is favourably regarded because it is oral, effective and well tolerated. Baricitinib was designed as a modification of tofacitinib that would inhibit JAK1 and JAK2 rather than JAK3.

Baricitinib has been tested in phase 2 and 3 studies leading up to registration in Europe in 2017 and USA and Australia in 2018. The main indication has been for rheumatoid arthritis with the primary outcome of trials being whether a 20% improvement in a clinical score was achieved (ACR20). These studies include determining the efficacy of baricitinib in patients with inadequate responses to methotrexate [18], comparing baricitinib with methotrexate in patients beginning therapy with disease-modifying agents (RA-BEGIN) [19], the use of baricitinib in patients who were intolerant to DMARDS (RA-Build) [20], baricitinib compared to anti-TNFα therapy (RA-BEAM) [13] and the efficacy of baricitinib in patients with moderate to severe RA with an inadequate response to anti-TNFα therapy (RA-Beacon) [12]. These studies concluded that baricitinib as a single agent is significantly more effective than methotrexate or anti-TNFα treatments and that this effect was seen with 4 mg/d of drug with effects also seen at 2 mg and at higher doses. Approximately 70% of participants achieved ACR20 with baricitinib alone.

Studies with baricitinib have also been carried out for inflammatory bowel disease (both ulcerative colitis and Crohn’s disease), psoriasis and psoriatic arthritis, atopic dermatitis [21], ankylosing spondylitis and systemic lupus erythematosus (SLE) [22, 23]. Tofacitinib has been approved for ulcerative colitis and psoriatic arthritis and a phase 2b dose escalation study of baricitinib showed positive results for psoriasis [24]. Baricitinib has shown efficacy in phase 2 and 3 trials for atopic dermatitis and has recently been approved in Europe for the treatment of moderate to severe disease in adult patients [25]. It has also been used in smaller studies and individual cases in alopecia areata [26], dermatomyositis [27], Candle syndrome and other interferonopathies [28]. More recently, the US Food and Drug Administration has provided Emergency Use Authorization for baricitinib in combination with remdesivir for the treatment of SARS-CoV-2 infections [16]. There continue to be ongoing studies, mostly sponsored by Eli Lilly, for atopic dermatitis in both adults (NCT03559270) and children (NCT03952559), SLE (NCT03843125), juvenile idiopathic arthritis (NCT03773965), giant cell arteritis (NCT03026504), pyoderma gangrenosum (NCT04901325) and hospitalization secondary to SARS-CoV-2 infection (NCT04421027).

### Objectives {7}

The primary objective is to assess whether baricitinib can reduce the loss of plasma C-peptide in patients with new onset T1D after 48 weeks of treatment. The study has several secondary objectives. Firstly, to assess the efficacy of baricitinib at additional timepoints on mitigating the loss of plasma C-peptide; secondly, to assess the effect of baricitinib on secondary clinical outcomes including insulin dose, haemoglobin A1c (HbA1c) and estimated C-peptide (CP_EST_) [29], a surrogate measure of beta cell function; and finally, to assess the safety and tolerability of baricitinib in participants with T1D. Additional exploratory objectives aim to establish the immune mechanism by which baricitinib impacts T1D and evaluate the impact of baricitinib on T1D-associated immune responses.

### Trial design {8}

The study is an investigator-initiated, randomized, two-arm (2:1, baricitinib to placebo), multicentre, double-blind, superiority, phase 2 clinical trial. The participants will be randomly assigned, in a 2:1 ratio, to either baricitinib for 48 weeks or placebo for 48 weeks and therefore will be followed for 96 weeks from the start of treatment.

## Methods: participants, interventions and outcomes

### Study setting {9}

The BANDIT trial is coordinated by the St Vincent’s Institute of Medical Research, Melbourne, Australia. Four tertiary centres in Australia will recruit and manage participants throughout the trial: St Vincent’s Hospital Melbourne, The Royal Melbourne Hospital, The Royal Children’s Hospital Melbourne and the Women’s and Children’s Hospital Adelaide. Data will be collected in Australia.

List of study sites can be accessed via the Australian New Zealand Clinical Trials Registry [ACTRN12620000239965].

### Eligibility criteria {10}

Participants will be deemed eligible by the treating physicians and nurses at the time of T1D presentation and diagnosis at each of the four tertiary hospitals.

#### Inclusion criteria

Participants must meet *all* of the following criteria to be eligible for the study:

- Male or female aged between 10 and 30 years (inclusive) at screening
- Diagnosis of T1D according to ADA criteria within 100 days prior to starting study drug
- Islet autoantibody positivity (one or more of: GADA, IA-2A, IAA (assessed within one week of commencing insulin therapy), ZnT8A)
- Stimulated (peak or 90 min) C-peptide >0.2 nM during a 2-h MMTT at the screening visit, or random C-peptide result >0.3 nM during the screening period
- Participants of childbearing age who are sexually active must agree to use of effective birth control until the end of the study
- Be able to read, understand and give written informed consent
- Be willing to comply with intensive diabetes management

#### Exclusion criteria

Participants must *not* meet any of the following criteria:

- Use of immunosuppressive or immunomodulatory therapies other than inhaled or topical glucocorticoids
- Current or past history of deep vein thrombosis or pulmonary embolism
- Impaired renal function defined by estimated glomerular filtration rate (according to the CKD-EPI) of < 60 mL/min/1.73 m^2^
- LDL cholesterol >4mmol/l
- Elevated liver function tests at screening
  - Aspartate aminotransferase 2x ULN
  - Alanine aminotransferase 2 x ULN
- Clinically significant abnormal laboratory parameters at screening including but not limited to:
  - Haemoglobin < 8 g/L
  - White blood cells <2500 cells/μl
  - Lymphocyte count <750 cells/μl
  - Platelets <50,000 cells/μl
  - Neutrophils <1200cells/μL
- Known hypersensitivity to baricitinib
- Known malignancy with the exception of successfully treated non-metastatic basal cell and squamous cell carcinoma
- Pregnancy, a desire for pregnancy, breast feeding, or a desire to father a child during the study
- Patients with current or recent (within 12 weeks of screening) clinically significant comorbidity, including clinically serious viral, bacterial, fungal, or parasitic infection. Viral infections include HBV, HCV, EBV, HIV, recent herpes zoster, and TB
- Treatment with any investigational product within 30 days or 5 half-lives (whichever is longer) prior to baseline visit, or concurrent participation in a clinical trial with an investigational product or device
- Experienced any of the following within 12 weeks of screening: myocardial infarction, unstable ischemic heart disease, stroke or New York Heart Association Stage IV heart failure
- Any serious medical condition within 4 weeks of screening which places the participant at an unacceptable risk if he or she were to participate in the study or confounds the ability to interpret data from the study, including, but not limited to, symptomatic cardiovascular, renal, respiratory, hepatic, gastrointestinal, endocrine, haematological and neurological conditions or psychiatric illness/social situations that would limit compliance with study requirements
- Have had any major surgery within 8 weeks prior to screening or will require major surgery during the study that, in the opinion of the investigator, would pose an unacceptable risk to the participant
- History of chronic alcohol abuse or IV drug abuse or other illicit drug abuse within 2 years prior to screening

### Who will take informed consent? {26a}

Participants will be approached about the trial during their initial admission for T1D once acute treatment has restored metabolic stability. The treating doctor will provide information about the trial. The research team will also provide written information in the form of a Participant Information and Consent Form (PICF). The participant will be encouraged to ask questions and take time to consider the information. Consent will be sought by the study team. Minors (<18 years) will be fully informed about the research and given the opportunity to ask questions and to discuss the research with family, friends and a family doctor. A parent or guardian will provide formal consent.

### Additional consent provisions for collection and use of participant data and biological specimens {26b}

Blood samples collected throughout the study will be processed, frozen and stored for analysis to achieve the primary, secondary and exploratory objectives. If the participant consents, some of the sample may be stored indefinitely for other future research studies at the central study repository. Before any blood samples can be used for other research studies, approval from the hospital’s Human Research Ethics Committee will be obtained. If participants do not give consent for their blood samples to be used for future research, the samples will be discarded after analysis of the study endpoints is complete.

### Interventions

#### Explanation for the choice of comparators {6b}

Participants will receive either 4 mg/day orally of baricitinib or placebo for a duration of 48 weeks. Both groups will receive intensive diabetes management aimed at achieving near-normal metabolic control. Participants from both groups will continue to manage their blood glucose using insulin as per standard-of-care. As such, placebo has been chosen as the comparator for baricitinib.

#### Intervention description {11a}

Participants will be instructed to take one tablet from a bottle at about the same time each day (one tablet of baricitinib (4mg/tablet) or one tablet of placebo), with or without food, for 48 weeks. Baricitinib and placebo will be identical tablets manufactured by Eli Lilly.

#### Criteria for discontinuing or modifying allocated interventions {11b}

Treatment will be discontinued at the end of the treatment period or in certain cases if there is development of an adverse event reaction to the investigational product. In this clinical trial, an adverse event (AE) is any occurrence or worsening of an undesirable or unintended sign, symptom or disease whether or not associated with the treatment. In most cases, the investigational product will be transiently discontinued until the problem is resolved and participants will remain in the study. If the adverse event is severe, it may prevent continuation of the participant in the study. Serious adverse events (SAEs) are untoward medical occurrences that result in any of the following outcomes: death; a life-threatening adverse event; inpatient hospitalization or prolongation of existing hospitalization, with the exception of hospitalization relating to initial diagnosis of T1D; incapacity to conduct normal life functions; and congenital anomaly/birth defects in the offspring of a study participant. SAEs that will lead to permanent discontinuation from trial intervention for an individual participant include GI perforation, baricitinib hypersensitivity and venous thromboembolism.

Participants may also discontinue trial treatment if the participant or legal guardian requests to discontinue trial intervention, or the investigator decides to discontinue a participant from the trial intervention in case the participant:

- Is pregnant
- Demonstrates significant non-compliance with the trial intervention
- Experiences a serious or intolerable adverse event such that continued trial intervention would not be in the best interest of the participant
- Develops, during the course of the trial, symptoms or conditions listed in the exclusion criteria
- Requires a medication that is prohibited by the protocol
- Requires early discontinuation for any other reason

In addition, the progress of the study will be monitored by the Trial Management Group (TMG), which will review safety recommendations from the Safety Review Committee (SRC) and make decisions regarding continuation, termination or modification of the study. The SRC will perform regular reviews based on enrolment numbers of safety data and review of all SAEs and will make recommendations to the TMG for continuing, modifying or stopping the trial.

Events for review by the SRC in this trial include:

- Any death except those assessed as not related to study treatment on review by the protocol chairs, the clinical trial physician and the coordinating principal investigator
- Two or more of the first 10 treated participants or ≥20% of all treated participants experience a clinically significant, drug-related adverse event resulting in the permanent discontinuation of study treatment
- Any gastrointestinal perforation
- Venous thromboembolism
- Evidence of serious opportunistic infection, including tuberculosis, or EBV-associated lymphoproliferative disorder
- Any unexpected, treatment-related SAE resulting in permanent treatment discontinuation and not related to glycaemic events

If the trial is terminated, the investigator may withdraw all trial participants from the trial treatment.

#### Strategies to improve adherence to interventions {11c}

The investigator will maintain adequate records of the disposition of the investigational product, including the date and quantity of drug that was received, the participants to whom drug was dispensed and an account of any destroyed drug. A drug-dispensing log will be kept current for each participant and will contain the identification of each participant and the date and quantity of drug dispensed and returned. Tablet counts will be used to assess participant compliance with daily doses of the study medication.

#### Relevant concomitant care permitted or prohibited during the trial {11d}

Combinations with other JAK inhibitors have not been studied and are not permitted. Participants who start a concomitant medication will be advised to contact the study team to review its potential effects on baricitinib metabolism. Those who require an emergency therapy will be encouraged to pursue the necessary clinical care and contact the study team within 24 h. Whenever possible, the recommendation will be to use concomitant medications that do not alter baricitinib levels.

#### Provisions for post-trial care {30}

Participants will be followed for 48 weeks after completion of the treatment period, including monitoring for any adverse events. Participants will be cared for by their local health care provider during and after completion of the study.

### Outcomes {12}

The primary endpoint of the study is plasma C-peptide area under the curve (AUC) over 2 h following a mixed meal tolerance test (MMTT) at 48 weeks.

The secondary endpoints of the study are:

- Change from baseline in 2-h MMTT-stimulated C-peptide AUC at weeks 12, 24, 72 and 96
- Change from baseline in mean daily insulin use over 7 consecutive days during the 2 weeks prior to the assessment at weeks 12, 24, 48, 72 and 96
- Change from baseline in glycosylated haemoglobin (HbA1c) levels at weeks 12, 24, 48, 72 and 96
- Number of participants with responder status at weeks 12, 24, 48, 72 and 96
- Change in estimated C-peptide (CP_EST_) [29] from baseline at weeks 12, 24, 48, 72 and 96
- Continuous glucose monitoring (CGM) at baseline, 12, 24, 48 and 96 weeks
- Frequency and severity of all adverse events.

The following mechanistic endpoints will be assessed at baseline, 12, 24, 48 and 96 weeks

- Analysis of total and antigen-specific T cells, including tetramer analysis
- Treg analysis

The following mechanistic endpoints will be assessed at baseline, 24 and 96 weeks

- Assessment of IFNγ, IL-21 and IL-7 induced STAT1, STAT3 and STAT5 phosphorylation respectively in whole blood
- PBMC analysis using flow cytometry, including markers for lineage, activation, exhaustion and cytokine signalling
- 10x Chromium single cell sequencing

### Participant timeline {13}

**Table:** 

	Screening		48 weeks on study drug											48 weeks of follow-up	
Study visit	**1**	**2**	**3**	**4**	**5**	**6**	**7**	**8**	**9**	**10**	**11**	**12**		**13**	**14**
Time required (h)	**1**	**3**	**1**	**1**	**1**	**1**	**3**	**1**	**1**	**1**	**1**	**3**		**3**	**3**
*Study week*	*−4 to −1*		*0*	*2*	*4*	*8*	*12*	*16*	*20*	*24*	*36*	*48*		*72*	*96*
*Study day*			*0*	*14*	*28*	*56*	*84*	*112*	*140*	*168*	*252*	*336*		*504*	*672*
*Visit window, days*				±3	±3	±7	±7	±7	±7	±7	±7	±7		±14	±14
Clinical history	X														
Full examination	X														
Short examination, weight		X	X	X	X	X	X	X	X	X	X	X		X	X
Safety review and adverse events reporting			X	X	X	X	X	X	X	X	X	X		X	X
Study drug provided			X		X	X	X	X	X	X	X				
Pregnancy testing (if applicable)	X		X	X	X	X	X	X	X	X	X	X		X	X
Review of insulin use and diabetes control			X	X	X	X	X	X	X	X	X	X		X	X
Continuous glucose monitoring		X					X			X		X		X	X
Telephone contact			Regularly throughout the entire study												
Blood sampling	X	X	X	X		X	X	X	X	X	X	X	X	X	X
Mixed meal tolerance test		X						X			X		X	X	X

### Modifications to visit logistics and schedule in response to COVID-19

In response to the COVID-19 pandemic, the BANDIT trial is following the current advice of the Australian Department of Health as well as hospital specific restrictions at each study site to best mitigate the risk of SARS-CoV-2 infections amongst the participants and trial staff.

Adjustments have been made to study visits in order to reduce social contact where possible. These include allowing for visits to be conducted remotely via telehealth instead of face-to-face, local as opposed to tertiary site pathology collection, and delivery of the study drug by courier. On-site attendance has been limited to essential in-person visits, including the initial screening and consent visits, and visits that involve trial endpoints that are not routinely available at local pathology centres, such as the Mixed Meal Tolerance Test. Flexibility with the time windows of these visits has been increased to make them more achievable. Standard COVID-19 screening by questions as advised by local authorities is being conducted prior to each face-to-face visit and the visit re-scheduled if necessary.

In addition, all participants are encouraged to receive the full course of an Australian Government approved COVID-19 vaccination if eligible.

### Sample size {14}

The sample size of 83 participants was selected based on estimates of 2-h AUC mean C-peptide obtained from prior studies and using standard power calculations for comparison of treated and placebo groups [30, 31].

Using standard equations for comparison of two means, a sample size of 50 baricitinib treated and 25 placebo treated participants with complete data is needed to provide 80% power to detect 45% increase in mean log(C-peptide+1) (0.306 vs. 0.445, sd of 0.2), in the treated group using a two-sample *T*-test at 0.05 level of significance (two-sided). With the expected 10% dropout rate, the number of participants recruited into the study will be increased to 83 (55 and 28 in the intervention and control arms respectively). Should the number of drop-outs exceed 10%, additional participants may be recruited.

### Recruitment {15}

Recruitment will primarily occur through diabetes clinics and diabetologist’s private practices. In addition, a significant emphasis will be placed upon using social media platforms as a means of online advertisements. Furthermore, the study will be publicized in press releases, on-line through JDRF, via targeted emails and letters through Diabetes Australia and through the National Diabetes Services Scheme (NDSS) that distributes diabetes treatment consumables through pharmacies. Additional publicity or advertising may be conducted, with appropriate ethics approvals.

### Assignment of interventions: allocation

#### Sequence generation {16a}

Stratified block randomization with variable block sizes of 3, 6 or 9 will be used to generate the randomization schedule. This will be done by an independent researcher prior to study initiation. Age (<21 and ≥21 years) will be used as a stratification factor.

#### Concealment mechanism {16b}

A blinded list will be provided for the study coordinators and investigators to assign participants. This will be a list of numbers, with no information on what treatment is assigned. The study coordinator or investigator will assign the next available randomization number from the age appropriate list and will complete a prescription form specifying the randomization number.

The randomization lists sent to the pharmacy at each site will contain the treatment assignments for each randomization number. The pharmacy will dispense the correct treatment for that number according to their randomization list.

#### Implementation {16c}

Randomization lists are generated by the trial statistician. Lists will then be provided to each site. Study coordinators or investigators responsible for enrolling participants will assign each participant to the next available randomization number from the appropriate age list.

### Assignment of interventions: blinding

#### Who will be blinded {17a}

Study participants, coordinators, investigators, study staff, sponsor and sponsor’s representatives will be blinded to assignment of participants.

#### Procedure for unblinding if needed {17b}

Unblinding prior to study completion will occur if a participant’s well-being is threatened and the investigator believes unblinding is necessary to provide appropriate medical care or to protect the participant.

Unblinding envelopes will be provided to each site. These envelopes will be stored securely by the Study Coordinator/ Principal Investigator in the study files. One envelope will be provided for each randomization number, and will contain the treatment assignment information (active drug or placebo) for that number, in case emergency unblinding is necessary.

## Data collection and management

### Plans for assessment and collection of outcomes {18a}

Trial data collected includes general assessments (i.e. vital signs, pregnancy testing), laboratory assessments using blood collection samples and diabetes-specific assessments, including records obtained from each participant’s glucose diary and records of insulin use. Trial data at each study visit will be collected by study site personnel and recorded using an electronic case report form (eCRF). The relevant personnel have received training on how to use this system. Each data point entered into the eCRF will be verifiable against an original source record, such as the medical record or other participant notes.

### Plans to promote participant retention and complete follow-up {18b}

Participation in the trial provides the benefit of intensive diabetes management and thus will ideally encourage retention and completion of the trial. In addition to frequent study visits, participants will also be regularly contacted by the study team outside of the normal visit schedule via scheduled telephone calls, which will take place during the treatment and follow-up period.

Phone contacts are scheduled so that participants have contact with the study team via visit or call approximately every 2–3 weeks during the first 24 weeks, every 3–5 weeks during weeks 25–48 and every 6–8 weeks during the follow-up period.

Participants who prematurely exit the study will still be encouraged to participate in regular follow-up visits, ensuring data will continue to be collected. If the participant does not consent to follow-up visits, they should complete an end-of-study visit, which will include general, laboratory and diabetes-specific assessments as per the schedule of assessments.

### Data management {19}

All trial data will be entered onto the eCRF. Each data point entered will be verifiable against an original source record, such as the medical record or other participant notes. The eCRF contains checks for data values.

Data management will be performed using Viedoc Electronic Data Capture (EDC) system to collect, store and maintain the study research data. Viedoc EDC system is a 21 CFR Part 11 compliant database that is web/cloud based and has automatic hourly data backups. On study completion, the data will be downloaded and securely stored on the sponsor’s server in compliance with the data management plan, which conforms to electronic data standards.

Viedoc EDC system enables multisite users to contribute via the internet to a secure database and allows the study coordinator to oversee all data entries. Access to the trial database is password protected and will be restricted to certain study team members, with different levels of access assigned by the study coordinator.

Data will be stored for at least 15 years in compliance with local guidelines (Australian code for responsible conduct of research, NHMRC, 2018). In addition, data obtained from minors will be stored for 7 years after a child’s 18th birthday, or 15 years after the end of the study—whichever date is latest. If trial sites have a longer retention period, this will be observed for data from that site. After this time, the participants identifying information at the institution will be permanently deleted from the computer system and any hard copies will be destroyed. If extended or unspecified consent is obtained for future research, then data may be kept indefinitely.

### Confidentiality {27}

Any identifying participant information obtained for the study will remain confidential. No personal information that can be linked to a participant, such as their name and address, will leave the clinic sites. Only the researchers at the clinic site will have access to the code that links a participant to their data, and all information will be coded before it leaves the clinic.

Information about participants may be obtained from their health records held at the clinic site and other health services, for the purpose of the research. By signing the consent form, participants agree to the study team accessing health records if they are relevant for the study.

In any publication and/or presentation, information will be provided in a grouped or de-identified format so that participants cannot be identified.

### Plans for collection, laboratory evaluation and storage of biological specimens for genetic or molecular analysis in this trial/future use {33}

Genomics data will be stored in a repository at the St Vincent’s Institute of Medical Research in compliance with SVI’s data management plan. Before genomics data can be accessed and used for future research studies, approval from the hospital’s Human Research Ethics Committee will be obtained. Access to the databank will be restricted to certain study team members and access assigned by the data custodian (Coordinating Principal Investigator).

Also see item 26b.

## Statistical methods

### Statistical methods for primary and secondary outcomes {20a}

All analyses will be performed using either SPSS 22 (IBM Corp. Armonk, NY, USA) or Stata15 (StataCorp LP, College Station, TX, USA). An experienced biostatistician will oversee data collection and data analysis. Primary analysis will be performed based on intention to treat (ITT) with sensitivity analysis as per protocol to confirm the robustness of the findings. In case of participants missing the follow-up assessment, appropriate imputation techniques will be employed in consultation with the study statistician. All continuous data will be tested for normality prior to the data analysis and appropriate transformations will be applied for skewed data. The C-peptide levels will be log transformed using log(C-peptide+1) as appropriate. ANOVA analysis will be used to examine the change in primary outcome between groups.

The baseline differences between study arms will be examined using *T*-test for continuous normally distributed data, while the Wilcoxon rank-sum test will be used for skewed or ordinal data and either chi-2 or Fisher’s exact for categorical data. Multilevel mixed effect model will be constructed to examine the impact of intervention on longitudinal changes in primary outcome while controlling for potential confounders including gender, baseline age and C-peptide level.

Secondary and exploratory outcomes will be assessed using either ANOVA or Fisher’s exact test for continuous and categorical data respectively. Multilevel mixed effect regression analysis will be used to examine longitudinal changes while controlling for potential confounders, either identified during the analysis or those of clinical relevance. The per-protocol analysis will be performed, including only those who received at least 50% of the treatment in order to confirm the robustness of the ITT results. Logistic regression will be used to examine the risk of developing adverse events (AE) or SAEs. Any deviations from the original data analysis plan will be documented and reported as appropriate.

### Interim analyses {21b}

An interim analysis will be conducted after the week 24 clinical data for the first 21 participants are complete and a report on the study feasibility and safety will be presented to the SRC and the TMG, who oversee all aspects of the trial and perform safety oversight activities.

Safety results will be listed, tabulated using descriptive statistics and plotted when appropriate. Parameters to be studied may include incidence of AEs, vital signs, laboratory parameters, physical examination findings, concomitant medications and other safety analyses as deemed clinically appropriate. For some safety parameters, results may be summarized and plotted separately for adults (participants who were 18 years or older at the time of randomization) and adolescents (participants who were at least 10 but not yet 18 at randomization). Analyses of AEs may include special categories of AEs, such as rashes, infections and autoimmune disorders. AEs may also be summarized by time of onset.

### Methods for additional analyses (e.g. subgroup analyses) {20b}

Not applicable as there are no subgroup analyses planned.

### Methods in analysis to handle protocol non-adherence and any statistical methods to handle missing data {20c}

The per-protocol analysis will include only those who received at least 50% of the treatment in order to confirm the robustness of the ITT results. In case of participants missing the follow-up assessment, appropriate imputation techniques will be employed in consultation with the study statistician. All continuous data will be tested for normality prior to the data analysis and appropriate transformations will be applied for skewed data.

### Plans to give access to the full protocol, participant-level data and statistical code {31c}

The datasets analysed during the current study and statistical code are available from the corresponding author on reasonable request, as is the full protocol.

## Oversight and monitoring

### Composition of the coordinating centre and trial steering committee {5d}

This multicentre trial has three overseeing committees:

- *Study chair and steering committee* receives periodic reports from the trial manager/coordinator on the progress of the study and interim data summaries. As appropriate, abstracts and manuscripts dealing with the progress of the trial shall also be directed by the study chair and steering committee according to the study’s publications and presentations policies. The steering committee will meet bi-monthly.
- *Trial Management Group (TMG)* comprises of the clinical trial statistician, the coordinating Principal Investigator, the clinical trial coordinator and the data manager. The TMG will oversee all aspects of the conduct of the trial and will perform safety oversight activities and/or act on advice from other individual(s) or groups providing safety oversight, including from the clinical trial physicians and the SRC. The TMG will meet weekly.
- *Safety Review Committee (SRC)* performs regular reviews of safety data and review of all SAEs and will make recommendations for continuing or stopping the trial. The SRC meeting schedule is based on defined enrolment milestones. The SRC members are independent from the sponsor and competing interests.

### Composition of the data monitoring committee, its role and reporting structure {21a}

Data monitoring is performed by a TMG sub-group consisting of the data manager, trial coordinator and clinical monitor with statistical support when required. Interim data summaries will be provided to the TMG and steering committee for review. The SRC performs regular independent monitoring of safety data and provides feedback to the TMG.

### Adverse event reporting and harms {22}

All investigators must report AEs, including expedited reports, in a timely fashion to their respective HREC in accordance with applicable regulations and guidelines. All AEs will be reported to the TMG in accordance with the study’s Adverse Event Monitoring Plan and current NHMRC recommendations. The investigator will grade their severity according to common toxicity criteria and will make a determination of their relationship to investigational product

SAEs must be reported by the site to the sponsor within 24 h of when the site was notified of the event. The site must also report SAEs to the HREC if applicable. All SAEs will be reported to Lilly Global Patient Safety by the sponsor, including event severity and causality assessment. Event outcome and other follow-up information regarding the treatment and resolution of the event will be obtained and reported by the site when available, if not known at the time the event is initially reported. The follow-up information should contain sufficient detail to allow for a complete medical assessment of the case and an independent determination of possible causality.

Adverse events will be reviewed by the sponsor. The TMG will conduct regular safety reviews approximately every 6 months (and otherwise as needed) of adverse events by treatment group assignment.

### Frequency and plans for auditing trial conduct {23}

This study and its conduct will be monitored according to ICH-GCP guidelines by contracted independent Clinical Research Associates (CRAs). CRAs will visit the sites during the study at regular intervals according to the study’s monitoring plan. Monitoring is focused on processes, documentation and data. Monitoring is not an audit of staff or teams and is not done to compare people to each other.

### Plans for communicating important protocol amendments to relevant parties (e.g. trial participants, ethical committees) {25}

No deviations from or changes to the protocol will be implemented without documented approval from the HREC of an amendment, except where necessary to eliminate an immediate hazard(s) to trial participants, or when the change(s) involves only logistical or administrative aspects of the trial. All necessary parties will be informed of any amendments made. Online trial registries will be updated accordingly.

### Dissemination plans {31a}

After all participants have completed the study and data are analysed, it is planned that the results will be disclosed completely and published in a medical journal with all data de-identified. Information about the trial results will also be published on the JDRF website.

## Discussion

This study is designed to evaluate the efficacy of baricitinib in slowing the progressive, immune-mediated loss of beta cell mass and function that occurs after clinical presentation of type 1 diabetes.

### Limitations

The maximum benefit of immunotherapy is most likely in individuals with pre-symptomatic (stage 1 or 2) T1D, where beta cell function is better preserved. Stage 3 (new-onset) trials like BANDIT currently provide opportunities to determine if new immunotherapies can preserve beta cell function and to identify treatment biomarkers as a prelude for testing in the more challenging stage 1 or 2 context.

### Strengths

This is the first human trial investigating the efficacy of JAK inhibitors in delaying the progression of T1D in both children and adults. This will provide important insights into understanding the importance of this pathway in the pathophysiology of T1D in humans, specifically the JAK1/JAK2 pathways. This will pioneer future exploration of the JAK-STAT pathway for T1D disease modification. The mechanistic studies conducted will play a key role in understanding the effects of baricitinib on general and beta cell specific immune responses and to determine if baricitinib-induced clinically relevant immunomodulatory effects can be detected and monitored.

## Trial status

The current working protocol is Version 6 of 03-06-2021. Recruitment of participants commenced in December 2020. At present, approximately 80 participants have been randomized to the trial. Recruitment is on track to finish in March 2022. Submission of this manuscript has been delayed due to insufficient resources and Covid-related modifications to the conduct of the study.

## Data Availability

On study completion, the eCRF data will be downloaded and securely stored on the SVI server in compliance with SVI’s data management plan, which conforms to electronic data standards. Access to the databank is restricted to certain study team members and access assigned by the data custodian (Coordinating Principal Investigator). The final trial data for this protocol can be supplied on request.

## Abbreviations

ADA: American Diabetes Association
AE: Adverse event
ALT: Alanine aminotransferase
ANOVA: Analysis of variance
AST: Aspartate aminotransferase
AUC: Area under the curve
CMV: Cytomegalovirus
CRF/eCRF: Case report form/electronic case report form
CGM: Continuous glucose monitoring
CKD-EPI: Chronic Kidney Disease Epidemiology Collaboration
CyTOF: Mass cytometry
DC: Dendritic cell
DCCT: Diabetes Control and Complications Trial
DKA: Diabetic ketoacidosis
DMSO: Dimethylsulfoxide
DVT: Deep vein thrombosis
EBV: Epstein-Barr virus
FCS: Foetal calf serum
GADA: Glutamic acid decarboxylase antibodies
GCP: Good Clinical Practice
GI: Gastrointestinal
HbA1c: Haemoglobin A1c
HDL: High-density lipoprotein
HepB/HBV: Hepatitis B/hepatitis B virus
HepC/HCV: Hepatitis C/hepatitis C virus
HIV: Human immunodeficiency virus
HLA: Human leukocyte antigen
HREC: Human Research Ethics Committee
IAA: Insulin autoantibodies
IA-2A: Insulinoma-associated-2 autoantibodies
ICH: International Conference on Harmonisation
IFN: Interferon
ITT: Intention to treat
JAK: Janus kinase
LDL: Low-density lipoprotein
LFT: Liver function test
MMTT: Mixed-meal tolerance test
NHMRC: National Health and Medical Research Council
NK cell: Natural killer cell
NOD: Nonobese diabetic
PBMC: Peripheral blood mononuclear cell
PI/CPI: Principal Investigator/Coordinating or Chief Principal Investigator
PICF: Patient information consent form
PLN: Pancreatic lymph node
RA: Rheumatoid arthritis
RGO: Research Governance Organization
SAE: Serious adverse event
SAP: Statistical analysis plan
SoA: Schedule of assessments
SRC: Safety Review Committee
STAT: Signal transducers and activators of transcription
SVI: St Vincent’s Institute
TB: Tuberculosis
T1D: Type 1 diabetes
TMG: Trial Management Group
ULN: Upper limit of normal
VZV: Varicella zoster virus
ZnT8: Zinc transporter 8
